# Strategic campaign attention to abortion before and after *Dobbs*

**DOI:** 10.1073/pnas.2503080122

**Published:** 2025-05-12

**Authors:** Mellissa Meisels

**Affiliations:** ^a^Department of Political Science, Yale University, New Haven, CT 06511

**Keywords:** elections, campaign agendas, abortion, congress, supreme court

## Abstract

In 2022, the US Supreme Court overturned the constitutional protection of abortion rights established in *Roe v. Wade*. In doing so, *Dobbs v. Jackson Women’s Health Organization* moved status quo on abortion policy more into line with the Republican Party’s stance. Subsequent research has documented the decision’s impact on mass political behavior and opinion, yet less is known about its impact on the behavior of political elites. I provide evidence on congressional candidates’ strategic responses to the decision with original data on campaign platforms (*N* = 4,703) from election cycles before and after *Dobbs*. After the decision, Democrats became significantly more likely to campaign on abortion and to do so using unambiguous language, while Republicans increasingly obfuscated their positions on the issue. Pre-post-*Dobbs* change in partisan divergence in campaign attention to abortion was driven most strongly by candidates in states with abortion bans set to take effect upon overturning of *Roe* (i.e., trigger laws and/or pre-*Roe* laws). Importantly, these shifting patterns of campaign attention were not present in other issue domains, consistent with changes in attention to abortion being driven by *Dobbs* rather than other contemporaneous factors.

By overturning their 1973 decision in *Roe v. Wade*, the US Supreme Court eliminated the federal right to abort in the first trimester, thereby allowing states to adopt sweeping abortion restrictions. The majority opinion in *Dobbs v. Jackson Women’s Health Organization* (2022), a draft of which was first leaked on May 2nd before the final decision was issued on June 24th, brought status quo on abortion policy closer to the Republican Party’s decades-long opposition to abortion. In doing so, *Dobbs* also constituted a rare case of the Supreme Court moving status quo away from majority public opinion, which supports legalized abortion access (1).

Subsequent research has explicated the aftermath of the decision vis-à-vis mass political behavior. For example, individuals perceived more widespread support for abortion afterward (2), and the presence of abortion-related measures on the ballot likely harmed Republicans in the 2022 midterms (3). Despite significant changes in the political environment induced by *Dobbs*, however, efforts to understand how it altered political elites’ strategic behavior have been far more elusive. Two key aspects of the decision have potential implications for candidates’ campaign strategies before versus after *Dobbs*.

First, the landmark case and its accompanying media coverage increased the salience and importance of the issue of abortion among the public (4). As a result, candidates likely faced greater pressure to campaign on abortion after *Dobbs* in order to appear responsive to voters’ top concerns (5, 6). Second, popular discourse and abortion-related ballot initiative outcomes in battleground and even Republican-controlled states subsequently revealed the extent of the unpopularity of the Republican stance on abortion relative to the Democratic stance (2). Given that candidates benefit from focusing on issues on which they enjoy an advantage and ignoring those on which they do not (7, 8), Democrats were likely incentivized to campaign more on abortion after *Dobbs* while Republicans were incentivized to campaign less. Taken together, Democrats likely faced straightforward incentives to increase attention to abortion while Republicans may have been cross-pressured.

I offer a systematic study of how legislative campaign strategy changed in response to *Dobbs*. Drawing on an original dataset of campaign platforms from elections before and after the decision, I show that candidates’ strategies diverged by party. While Democrats became substantially more likely to campaign on abortion and to do so using unambiguous language, Republicans increasingly obfuscated their abortion positions. Moreover, the pre-post-*Dobbs* change in partisan divergence in campaign attention to abortion was concentrated most strongly among candidates running in states with abortion bans set to take effect upon overturning of *Roe*. There were no analogous changes in the same candidates’ attention to other issues before and after *Dobbs*, providing further evidence that these results are driven by domain-specific effects of the decision.

## Materials and Methods

I collect all available issue platforms found on House primary candidates’ campaign websites from 2016 to 2024 (9), allowing for the identification of issues each candidate chose to campaign upon in a particular election. The Court first granted certiorari in the *Dobbs* case in 2021 and a draft of the majority opinion leaked in May 2022, after primaries in some but not all states had been held. As such, the dataset includes campaign platforms from three fully pre-*Dobbs* elections (2016, 2018, 2020), the partially “treated” election of 2022, and the fully post-*Dobbs* election of 2024. I identify whether a campaign platform devotes attention to abortion and seven other issue areas by performing simple string-matching with collections of terms associated with each issue (10).^*^SI Appendix includes discussion of some nonabortion issue domains, as well as how their trends may provide further insight into how candidates’ strategies responded to different aspects of *Dobbs*.

**Fig. 1.**
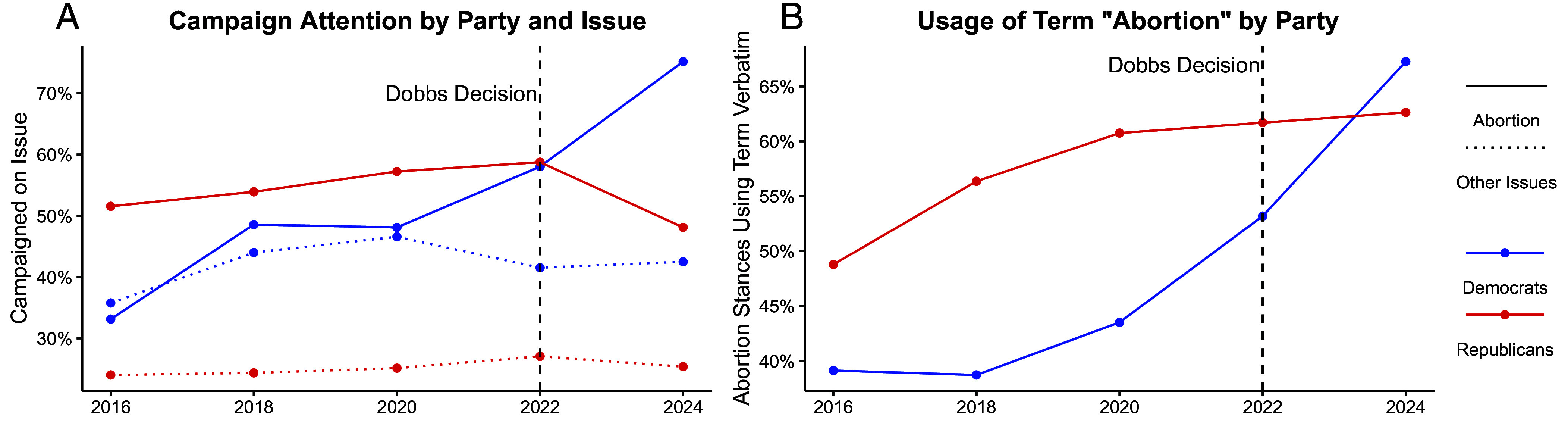
(*A*) US House candidates’ platforms across time show more Democrats and fewer Republicans campaigning on abortion after *Dobbs*, while there was little contemporaneous partisan divergence in campaign attention to other issues. (*B*) The share of Democrats using the term “abortion” verbatim when campaigning on the issue also rose sharply after *Dobbs*.

**Fig. 2.**
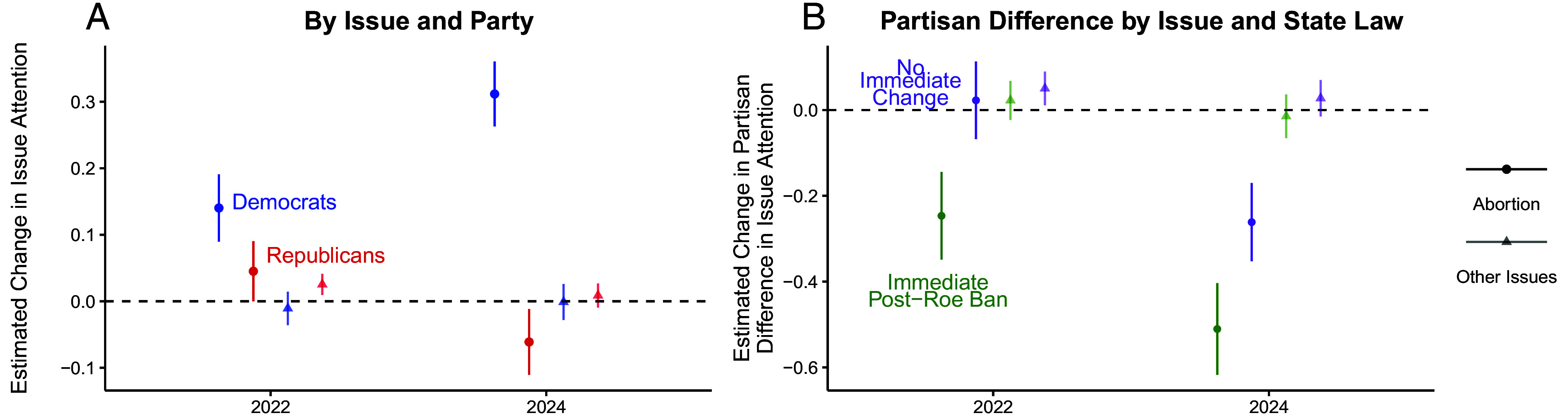
(*A*) Pre-post-*Dobbs* change in House candidates’ likelihood of campaigning on abortion vs. other issues by party. Democrats became significantly more likely to campaign on abortion, but not other issues, compared to Republicans. (*B*) Pre-post-*Dobbs* change in partisan difference in likelihood of campaigning on abortion vs. other issues by candidates running in states with trigger laws and/or pre-*Roe* abortion bans and those running in states without. After *Dobbs*, the partisan divergence grew significantly more in states with an immediate abortion ban than in states without. Coefficients from linear probability models with 95% CI based on candidate-clustered SE are shown.

Fig. 1*A* plots the share of Democrats’ and Republicans’ primary campaign platforms which include the issue of abortion in each election cycle, compared to the same platforms’ averaged attention across the seven other issues. Fig. 1*B* shows the share of platforms which use the term abortion verbatim among candidates who chose to campaign on abortion. Fig. 2*A* plots coefficients and corresponding 95% CI from linear probability models estimating candidates’ decision to campaign an issue in 2022 and 2024 compared to pre-*Dobbs* elections (i.e., 2016 to 2020) separately by party and abortion versus other issues. Fig. 2*B* plots coefficients and corresponding 95% CI from models analogous to those in Fig. 2*A* but interacting the year indicators with candidate party and performing estimation separately by abortion versus other issues and whether candidates ran in states with pre-*Roe* abortion bans or trigger laws.^†^

## Partisan Divergence in Post-Dobbs Campaign Attention to Abortion

After *Dobbs*, the proportion of Democrats campaigning on abortion increased while Republicans’ share decreased (Fig. 1*A*). In elections prior to 2022, Republicans consistently campaigned on abortion more than Democrats. In 2022, however, the same shares of each party’s candidates campaigned on the issue. By 2024—after the unpopularity of Republicans’ abortion stance had been further revealed by the outcomes of state ballot propositions—the share of Republicans campaigning on abortion actually declined, marking the first time since before 2016 that fewer than half of Republican candidates campaigned on the issue.

Trends in campaign attention to other issues over the same period provide some reassurance that these changes in campaign attention to abortion are due to *Dobbs*. In theory, it could be the case that Democrats’ campaigns became more issue-oriented in 2022 and 2024 while Republicans’ campaigns became less issue-oriented in 2024. This would lead partisans’ campaign strategies on abortion to diverge post-*Dobbs* for reasons which need not be related to the decision. However, we do not observe analogous changes in average attention to other issues in the same campaign platforms over the same period, suggesting domain-specific changes.

Moreover, the substance of campaign rhetoric related to abortion also changed after *Dobbs*. While Democrats overwhelmingly opted to use more euphemistic terms (e.g., “choice” and “reproductive rights”) when campaigning on abortion before the decision, by 2024 usage of the term abortion actually became more prevalent among Democrats who campaigned on the issue than Republicans who campaigned on the issue (Fig. 1*B*).

The magnitude of these changes is estimated more formally in Fig. 2*A*. Democrats were 14 percentage points more likely to campaign on abortion in 2022 and 31 percentage points more likely in 2024 compared to pre-*Dobbs* elections (both *P* < 0.01, Fig. 2*A*). They were not, however, any more likely to campaign on nonabortion issues in either 2022 or 2024. In contrast, Republicans became significantly less likely to campaign on abortion by 2024 (*P* < 0.05), and while they appeared somewhat more likely to campaign on abortion in 2022 (*P* = 0.051), they were also more likely to campaign on other issues that year (*P* = 0.015).

Next, I estimate post-*Dobbs* changes in partisan difference in campaign attention to abortion by whether candidates ran in states with post-Roe abortion ban laws in place (i.e., trigger laws and/or pre-*Roe* abortion bans). Interacting Republican identification with year indicators, Fig. 2*B* shows that in states with automatic bans in place, Republicans became significantly less likely to campaign on abortion after *Dobbs* compared to Democrats (*P* < 0.01, 2022 and 2024), while there was no comparable change on average in other issue domains (*P* > 0.10, 2022 and 2024). In states without trigger laws or pre-*Roe* bans, a substantial change in this partisan difference in likelihood of campaigning on abortion did not occur until the 2024 election, and the difference still remained significantly larger in automatic ban states.

## Discussion

Previous research suggests that *Dobbs* harmed Republicans and benefitted Democrats in 2022, yet little is known about whether or how candidates systematically adapted to the new political environment imposed by the decision. This study demonstrates that Democrats increasingly devoted campaign attention to the issue of abortion after the decision, while Republicans obfuscated on the issue.

These patterns of change in partisan divergence are consistent with a number of potential mechanisms. The *Dobbs* decision substantially altered status quo policy on abortion, which both increased the salience of the issue and increased Democrats’ perceived advantage on the issue given public backlash to the Republican-aligned new status quo. Salience alone cannot explain the results, as both Democrats and Republicans would have focused more on abortion following the decision. The results may therefore be driven by public opinion considerations, with Democrats having sought to capitalize on the newly realized popularity of their position on abortion and Republicans attempting to minimize backlash to their position by obfuscating on the issue.

On the other hand, these campaigning changes may have been driven by changes in candidates’ relative satisfaction with the status quo, as Republicans were better off and Democrats worse off after *Dobbs*. However, timing of the changes suggests that this may not have been the case. Republicans did not become less likely to campaign on abortion until 2024, after the unpopularity of their position had been further revealed by state ballot propositions, while their satisfaction with the status quo would have increased immediately following the decision in 2022.

At a time when abortion became an especially important issue to the public (4), discordant campaign strategies may have made it especially challenging for them to compare candidates’ positions on the issue. Although voters could have attempted to infer candidates’ positions using a party heuristic in theory, in practice many are unaware of where parties fall on either side of even major issues (11). Moreover, such cues are unhelpful for distinguishing between candidates in intraparty or nonpartisan settings such as primaries, which are becoming increasingly consequential for the outcome of House elections (12). When estimating mass behavioral effects of unpopular policy changes, scholars should also consider the upstream incentives which shape political elites’ equilibrium behavior.

## Supplementary Material

Appendix 01 (PDF)

## Data Availability

R data and code files to reproduce results are available via Harvard Dataverse (13). *SI Appendix* includes further technical details.

## Footnotes

^*^Other issues include Guns, Environment, Animal Rights, Police, Elderly, LGBTQ, and Campaign Finance. Israel, another issue area previously identified in campaign platforms, is excluded due to the attack on October 7, 2023, likely shifting the domain-specific status quo. Empirically, however, results are unchanged when including the issue as both Democrats and Republicans slightly increased campaign attention to Israel between 2022 and 2024.

^†^The following states had trigger laws or pre-*Roe* bans on abortion: LA, ID, WY, SD, ND, UT, AZ, TX, OK, AR, AL, GA, NC, TN, KY, WV, MO, WI, and MI.
