# Supplementary material for: Strategic campaign attention to abortion before and after Dobbs
Source: Proc Natl Acad Sci U S A. 2025 May 12;122(20):e2503080122. doi: 10.1073/pnas.2503080122 (PMC12107182; doi:10.1073/pnas.2503080122)
Supplement: Supplementary file 1 — Appendix 01 (PDF) [file pnas.2503080122.sapp.pdf]

Supporting Information for

Strategic campaign attention to abortion before and after *Dobbs*

Mellissa Meisels

E-mail: mellissa.meisels@yale.edu

This PDF file includes:

Supporting text

Supporting Information Text

Model Details

Figure 2A. Four separate models were estimated by party and for the issue of abortion and non-abortion issues. The outcome was whether the candidate campaigned on the issue that year, modeled as a function of whether the year was 2022 or 2024, with the reference time category being before 2022 (i.e. 2016, 2018, or 2020). Standard errors were clustered at the candidate level.

Figure 2B. Four separate models were estimated by pre-*Dobbs* state law status and for the issue of abortion and non-abortion issues. The outcome was whether the candidate campaigned on the issue that year, modeled as a function of whether the year was 2022 or 2024, with the omitted time category being before 2022 (i.e. 2016, 2018, or 2020), and interactions between an indicator for whether the candidate was a Republican and the year dummies. Standard errors were clustered at the candidate level. Figure 2B plots the interaction coefficients to capture the estimated partisan difference in the change from before *Dobbs* to 2022 and 2024.

Trends in Other Issue Domains

I include results from the same candidates' attention to non–abortion issues over the same period to serve as a "baseline" for general secular changes in campaign attention. However, on an issue-by-issue basis, there is a spectrum of how much the domain-specific political environment changed over time. Given that *Dobbs* bundled changes in status quo policy with changes in issue salience, considering trends in other issue domains which only experienced change in one or the other over the period is useful for considering which aspect of the decision likely induced changes in candidates' behavior.

For example, the non-abortion issue with the most substantial change to partisan difference in campaign attention over the period was policing. Prior to 2022, a greater share of Democrats campaigned on the issue than Republicans, after which Democrats lost their "ownership" of policing. Like abortion, the salience of policing issues increased substantially after 2020, not because of a top-down status quo policy shift but because of high-profile shootings by police officers and large-scale Black Lives Matter protests. Changes in partisan attention to policing may be consistent with Republicans' desire to capitalize on the increased salience of policing by attempting to align Democrats with recent unpopular developments in the domain, such as calls to "defund the police" and protest-related incidents of crime.

These patterns in the domain of policing suggest that changes to partisan differences in campaigning are a combination of changes in increased salience and perceived advantage on an issue, rather than a status quo policy shift *per se*. In the case of abortion, *Dobbs* both shifted status quo policy and increased issue salience, whereas policing salience was increased from the bottom up. Trends in both cases are consistent with one party believing to have an advantage on the issue's newfound salience.

Additionally, I exclude the issue of Israel from analyses because of Hamas' attack and the ensuing war potentially constituting a status quo shift. However, because of actions on both sides, it is likely ambiguous whether status quo was moved further from or closer to public opinion on the issue. This is consistent with patterns in the data: between 2022 and 2024, there was a slight increase in both Democrats' and Republicans' attention to the issue, resulting in very little change in partisan difference. This means that, in practice, the exclusion of Israel from the set of "control" issues is inconsequential.

Term Dictionaries

The following terms were string matched to campaign platforms to identify whether or not candidates campaigned on each issue.

Abortion. "sanctity of life", "unborn", "pro-life", "fetus", "abortion", "abort", "naral", "global gag", "planned parenthood", "terminate", "rape", "right to life", "right to choose", "pro-choice", "pregnancy", "roe", "hyde", "family planning", "reproductive"

Guns. "2nd amendment", "infringe", "right to bear arms", "militia", "second amendment", "self-defense", "nra", "rifle", "rifles", "ammunition", "firearm", "firearms", "gun violence", "shooting", "shootings", "shooter", "assault rifle", "automatic rifle", "automatic rifles", "automatic weapons", "assault weapon", "automatic weapon", "background checks", "background check", "bump stock", "high-capacity magazine", "gun", "guns", "high-capacity magazines"

Animals. "animal","animals","pet", "pets"

LGBTQ. "religious freedom", "marriage equality", "traditional marriage", "same sex marriage", "same-sex marriage", "traditional marriages", "same-sex marriage", "gay", "same sex", "same-sex", "sexual orientation", "lgbt+", "lgbt", "lgbtq", "lgbtq+", "transgend", "sanctity of marriage", "conversion therapy", "gender affirming", "gender-affirming"

Elderly. "senior", "seniors", "retiring", "retired", "retire", "retires", "retirees", "retirement", "older americans", "old-age", "old age"

Campaign Finance. "citizens united", "campaign finance", "financial disclosure"

Environment. "clean energy", "environment","environmental", "climate change", "global warming", "greenhouse", "pollution", "polluting", "pollutants", "polluters", "fossil fuel", "fossil fuels", "carbon", "clean fuel", "ecosystem", "planet", "solar energy", "solar panels"

Police. "defund the police", "abolish", "law enforcement", "policing", "back the blue", "police", "protect and serve", "profiling", "incarcer", "officer", "officers"
